# Interim Estimates of 2019–20 Seasonal Influenza Vaccine Effectiveness — United States, February 2020

**DOI:** 10.15585/mmwr.mm6907a1

**Published:** 2020-02-21

**Authors:** Fatimah S. Dawood, Jessie R. Chung, Sara S. Kim, Richard K. Zimmerman, Mary Patricia Nowalk, Michael L. Jackson, Lisa A. Jackson, Arnold S. Monto, Emily T. Martin, Edward A. Belongia, Huong Q. McLean, Manjusha Gaglani, Kayan Dunnigan, Angie Foust, Wendy Sessions, Juliana DaSilva, Shoshona Le, Thomas Stark, Rebecca J. Kondor, John R. Barnes, David E. Wentworth, Lynnette Brammer, Alicia M. Fry, Manish M. Patel, Brendan Flannery

**Affiliations:** ^1^Influenza Division, National Center for Immunization and Respiratory Diseases, CDC; ^2^University of Pittsburgh Schools of the Health Sciences and University of Pittsburgh Medical Center, Pittsburgh, Pennsylvania; ^3^Kaiser Permanente Washington Health Research Institute, Seattle, Washington; ^4^University of Michigan School of Public Health, Ann Arbor, Michigan; ^5^Marshfield Clinic Research Institute, Marshfield, Wisconsin; ^6^Baylor Scott & White Health, Texas A&M University College of Medicine, Temple, Texas.

During the 2019–20 influenza season, influenza-like illness (ILI)* activity first exceeded the national baseline during the week ending November 9, 2019, signaling the earliest start to the influenza season since the 2009 influenza A(H1N1) pandemic. Activity remains elevated as of mid-February 2020. In the United States, annual vaccination against seasonal influenza is recommended for all persons aged ≥6 months (*1*). During each influenza season, CDC estimates seasonal influenza vaccine effectiveness in preventing laboratory-confirmed influenza associated with medically attended acute respiratory illness (ARI). This interim report used data from 4,112 children and adults enrolled in the U.S. Influenza Vaccine Effectiveness Network (U.S. Flu VE Network) during October 23, 2019–January 25, 2020. Overall, vaccine effectiveness (VE) against any influenza virus associated with medically attended ARI was 45% (95% confidence interval [CI] = 36%–53%). VE was estimated to be 50% (95% CI = 39%–59%) against influenza B/Victoria viruses and 37% (95% CI = 19%–52%) against influenza A(H1N1)pdm09, indicating that vaccine has significantly reduced medical visits associated with influenza so far this season. Notably, vaccination provided substantial protection (VE = 55%; 95% CI = 42%–65%) among children and adolescents aged 6 months–17 years. Interim VE estimates are consistent with those from previous seasons, ranging from 40%–60% when influenza vaccines were antigenically matched to circulating viruses. CDC recommends that health care providers continue to administer influenza vaccine to persons aged ≥6 months because influenza activity is ongoing, and the vaccine can still prevent illness, hospitalization, and death associated with currently circulating influenza viruses as well as other influenza viruses that might circulate later in the season.

Methods used by the U.S. Flu VE Network have been published previously (*2*). At five study sites (Michigan, Pennsylvania, Texas, Washington, and Wisconsin), patients aged ≥6 months seeking outpatient medical care for an ARI with cough within 7 days of illness onset were enrolled once local influenza circulation was identified.^†^ Enrollment eligibility criteria included 1) age ≥6 months on September 1, 2019 (i.e., vaccine-eligible); 2) ARI with cough, with onset ≤7 days earlier; and 3) no treatment with influenza antiviral medication (e.g., oseltamivir or baloxavir) during this illness. Consenting participants or their proxies were interviewed to collect demographic data, information on general and current health status and symptoms, and 2019–20 influenza vaccination status. Nasal and oropharyngeal swabs (nasal swabs alone for children aged <2 years) were collected to obtain respiratory specimens; swabs were placed in a single cryovial with viral transport medium and tested at U.S. Flu VE Network laboratories using CDC’s real-time reverse transcription–polymerase chain reaction (RT-PCR) protocol for detection and identification of influenza viruses.^§^ For interim estimates, participants (including children aged <9 years, who require 2 vaccine doses during their first vaccination season) were considered to be vaccinated if they received ≥1 dose of any seasonal influenza vaccine ≥14 days before illness onset, according to medical records, registries, or patient report. VE against all influenza virus types combined and against viruses by type/subtype was estimated as 100% x (1 − odds ratio).^¶^ Estimates were adjusted for study site, age group, sex, race/ethnicity, self-rated health status, days from illness onset to enrollment, and month of illness using logistic regression. VE estimates by age group and influenza subtype are presented for strata with sufficient numbers of influenza cases to achieve adequate statistical power to detect a significant VE based on a priori sample size calculations.**

Among 4,112 ARI patients enrolled during October 23, 2019–January 25, 2020, a total of 1,060 (26%) tested positive for influenza virus infection by real-time RT-PCR, including 691 (17%) for influenza B viruses and 374 (9%) for influenza A viruses (Table 1); five patients tested positive for both influenza A and B viruses. Of 673 influenza B viruses with lineage information available, 670 (>99%) belonged to the B/Victoria lineage, and three (<1%) belonged to the B/Yamagata lineage. Among 335 subtyped influenza A viruses, 326 (97%) were A(H1N1)pdm09 viruses, and only 11 (3%) were A(H3N2) viruses. The proportion of patients with influenza differed among study sites, age groups, racial/ethnic groups, self-rated health status, and days from illness onset to enrollment. The percentage of ARI patients who were vaccinated ranged from 38% to 61% among study sites and differed by study site, sex, age group, race/ethnicity, self-rated health status, and days from illness onset to enrollment.

**TABLE 1:** Influenza real-time reverse transcription–polymerase chain reaction test results and seasonal vaccination status among patients with medically attended acute respiratory illness (N = 4,112), by selected characteristics — U.S. Influenza Vaccine Effectiveness Network, October 23, 2019—January 25, 2020

Characteristic	Test result status			Total no. of patients	Vaccinated no. (%)^†^	P-value*
	Influenza-positive no. (%)	Influenza-negative no. (%)	P-value*			
**Overall**	**1,060 (26)**	**3,052 (74)**	**N/A**	**4,112**	**2,072 (50)**	**N/A**
**Study site**						
Michigan	94 (25)	280 (75)	0.001	374	226 (60)	<0.001
Pennsylvania	222 (32)	466 (68)		688	346 (50)	
Texas	303 (25)	916 (75)		1,219	469 (38)	
Washington	236 (23)	787 (77)		1,023	620 (61)	
Wisconsin	205 (25)	603 (75)		808	411 (51)	
**Sex**						
Male	448 (27)	1,198 (73)	0.08	1,646	789 (48)	0.01
Female	612 (25)	1,854 (75)		2,466	1,283 (52)	
**Age group**						
6 mos–8 yrs	263 (29)	652 (71)	<0.001	915	470 (51)	<0.001
9–17 yrs	199 (41)	282 (59)		481	164 (34)	
18–49 yrs	413 (28)	1,084 (72)		1,497	595 (40)	
50–64 yrs	113 (18)	532 (82)		645	372 (58)	
≥65 yrs	72 (13)	502 (87)		574	471 (82)	
**Race/Ethnicity^§^**						
White	691 (24)	2,169 (76)	0.002	2,860	1,522 (53)	<0.001
Black	120 (32)	260 (68)		380	134 (35)	
Other race	111 (28)	291 (72)		402	227 (56)	
Hispanic	137 (30)	325 (70)		462	186 (40)	
**Self-rated health status^¶^**						
Fair or poor	55 (18)	248 (82)	<0.001	303	182 (60)	<0.001
Good	231 (21)	866 (79)		1,097	576 (53)	
Very good	393 (26)	1,141 (74)		1,534	761 (50)	
Excellent	380 (32)	794 (68)		1,174	549 (47)	
**Illness onset to enrollment (days)**						
<3	492 (35)	900 (65)	<0.001	1,392	653 (47)	<0.001
3–4	390 (26)	1,099 (74)		1,489	713 (48)	
5–7	178 (14)	1,053 (86)		1,231	706 (57)	
**Influenza test result**						
**Negative**	**N/A**	**3,052 (74)**	N/A	**3,052**	**1,682 (55)**	N/A
Influenza B positive**	**691 (17)**	**N/A**		**691**	**232 (34)**	
B/Yamagata	3 (<1)	N/A		3	3 (100)	
B/Victoria	670 (93)	N/A		670	221 (33)	
B lineage undetermined	18 (7)	N/A		18	8 (44)	
Influenza A positive**	**374 (9)**	**N/A**		**374**	**161 (43)**	
A (H1N1)pdm09	326 (63)	N/A		326	138 (42)	
A (H3N2)	11 (3)	N/A		11	7 (64)	
A subtype undetermined	39 (34)	N/A		39	16 (41)	

**Abbreviation:** N/A = not applicable.

* The chi-squared statistic was used to assess differences between the numbers of persons with influenza-negative and influenza-positive test results, in the distribution of enrolled patient and illness characteristics, and in differences between groups in the percentage vaccinated.

^†^ Defined as having received ≥1 dose of influenza vaccine ≥14 days before illness onset. A total of 104 participants who received the vaccine ≤13 days before illness onset were excluded from the study sample.

^§^ Patients were categorized into one of four mutually exclusive racial/ethnic populations: white, black, other race, and Hispanic. Persons identifying as Hispanic might have been of any race. Persons identifying as white, black, or other race were non-Hispanic. Race/ethnicity was missing for eight patients.

^¶^ General self-rated health status was missing for four patients.

** Five patients had coinfection with influenza A and influenza B, making the sum 1,065, or five more than the total number of influenza-positive patients. Two patients had coinfection with influenza A(H1N1)pdm09 and A(H3N2).

Among influenza-positive participants, 37% had received the 2019–20 seasonal influenza vaccine, compared with 55% of influenza-negative participants (Table 2). Overall, the adjusted VE was 45% against influenza A and B virus types combined, 50% against influenza B/Victoria, and 37% against A(H1N1)pdm09. VE was higher among children and adolescents aged 6 months–17 years and lower among adults aged 18–49 years, especially against A(H1N1)pdm09 (VE = 5%; 95% CI = -45% to 37%).

**TABLE 2:** Number and percentage of outpatients with acute respiratory illness and cough (N = 4,112) receiving 2019–20 seasonal influenza vaccine, by influenza real-time reverse transcription–polymerase chain reaction (RT-PCR) test result status, age group, and vaccine effectiveness* against all influenza A and B, B/Victoria and A(H1N1)pdm09 — U.S. Influenza Vaccine Effectiveness Network, October 23, 2019–January 25, 2020

Influenza type/Age group	Influenza-positive		Influenza-negative		Vaccine effectiveness	
	Total	Vaccinated no. (%)	Total	Vaccinated no. (%)	Unadjusted % (95% CI)	Adjusted^†^ % (95% CI)
**Influenza A and B**						
**Overall**	**1,060**	**390 (37)**	**3,052**	**1,682 (55)**	**53 (45 to 59)**	**45 (36 to 53)**
**Age group**						
6 mos–17 yrs	462	142 (31)	934	492 (53)	60 (50 to 69)	55 (42 to 65)
18–49 yrs	413	143 (35)	1,084	452 (42)	26 (6 to 42)	25 (3 to 41)
≥50 yrs	185	105 (57)	1,034	738 (71)	47 (27 to 62)	43 (19 to 60)
**Influenza B/Victoria**						
**Overall**	**634**	**211 (33)**	**2,968**	**1,641 (55)**	**60 (52 to 66)**	**50 (39 to 59)**
**Age group**						
6 mos–17 yrs	353	104 (29)	934	492 (53)	62 (51 to 71)	56 (42 to 67)
≥18 yrs	317	117 (37)	2,118	1,190 (56)	54 (42 to 64)	32 (11 to 48)
**Influenza A(H1N1)pdm09**						
**Overall**	**326**	**138 (42)**	**3,052**	**1,682 (55)**	**40 (25 to 53)**	**37 (19 to 52)**
**Age group**						
6 mos–17 yrs	98	35 (36)	934	492 (53)	50 (23 to 68)	51 (22 to 69)
18–49 yrs	125	48 (38)	1,084	452 (42)	13 (**−**27 to 40)	5 (**−**45 to 37)
≥50 yrs	103	55 (53)	1,034	738 (71)	54 (31 to 69)	50 (20 to 68)

* Vaccine effectiveness was estimated as 100% x (1 − odds ratio [ratio of odds of being vaccinated among outpatients with CDC’s real-time RT-PCR influenza-positive test results to the odds of being vaccinated among outpatients with influenza-negative test results]); odds ratios were estimated using logistic regression.

^†^ Adjusted for study site, age group, sex, race/ethnicity, self-rated general health, number of days from illness onset to enrollment, and month of illness using logistic regression.

As of January 25, 2020, CDC had genetically characterized 177 influenza B/Victoria viruses from U.S. Flu VE Network participants; 172 (97%) belonged to genetic subclade V1A.3, a different subclade from the V1A.1 subclade that includes the 2019–20 B/Victoria vaccine reference strain (B/Colorado/06/2017), and five (3%) belonged to V1A.1. All of the 32 genetically characterized A(H1N1)pdm09 viruses were from genetic group 6B.1A, which includes the 2019–20 A(H1N1)pdm09 vaccine reference strain (A/Brisbane/02/2018).

## Discussion

The 2019–20 influenza season began early with predominant influenza B/Victoria virus circulation, followed by increasing A(H1N1)pdm09 virus activity, with ongoing detection of both viruses (*3*). Through the week ending February 8, 2020, influenza activity remained elevated in most parts of the country (https://www.cdc.gov/flu/weekly). Markers of severe illness, including laboratory-confirmed influenza-associated hospitalization rates among children and adolescents aged <18 years and young adults aged 18–49 years, are higher than at this time in recent seasons, including the 2017–18 season. To date for this season, 92 influenza-associated deaths have been reported in children and adolescents aged <18 years; other than the 2009 pandemic, this is the largest number reported for this time of the season since reporting began for the 2004–05 influenza season (https://www.cdc.gov/flu/weekly). These interim VE estimates indicating a 45% reduction in influenza illness associated with a medical visit so far this season are particularly important in the context of the substantial prevalence of influenza in the United States: during the previous decade, influenza caused an estimated 4.3–21 million doctor visits, 140,000–810,000 hospitalizations, and 12,000–61,000 deaths each year.^††^

Among U.S. Flu VE Network participants, influenza virus infections accounted for approximately 25% of medically attended visits for ARI, demonstrating the considerable contribution of influenza virus infections to medically attended outpatient visits for ILI this season. Both influenza A and B viruses can cause severe illness, including hospitalizations and deaths. Some studies have suggested that influenza B virus infections might also result in more severe illness among children (*4*,*5*). Interim VE estimates indicate that the 2019–20 influenza vaccine protects against the predominant B/Victoria viruses from subclade V1A.3 and are consistent with VE estimates against influenza B/Victoria (range = 49%–56%) during seasons when the B/Victoria component of the vaccine was well matched to circulating viruses.^§§^

Influenza A(H1N1)pdm09 circulation increased in late December 2019; as of January 31, 2020, all A(H1N1)pdm09 viruses antigenically characterized at CDC were similar to the cell-propagated vaccine reference virus for the A(H1N1)pdm09 component of the 2019–20 Northern Hemisphere vaccine. Interim VE estimates against influenza A(H1N1)pdm09 viruses among children and older adults are consistent with average VE for influenza A(H1N1)pdm09 viruses reported previously (*6*). Among adults aged 18–49 years, the interim VE estimate against influenza A(H1N1)pdm09 was low and not statistically significant. Additional enrollment during the season while A(H1N1)pdm09 viruses circulate will determine whether VE against A(H1N1)pdm09 in this age group is lower than during previous seasons and will help evaluate potential contributing factors to lower than expected effectiveness.

During the five previous influenza seasons, the number of weeks that ILI activity was above baseline ranged from 11 to 20 weeks, with an average of 18 weeks (*7*). At 21 weeks, the 2018–19 influenza season was prolonged, demonstrating that influenza activity can continue beyond the winter months. CDC continues to recommend influenza vaccination while influenza viruses are circulating. Vaccination can protect against infection with influenza viruses that are currently circulating and those that might circulate later in the season. During the 2018–19 influenza season, in which influenza A(H3N2) and A(H1N1)pdm09 viruses cocirculated, interim VE was estimated to be 29% against illnesses associated with any influenza virus (*8*) and vaccination was estimated to prevent 4.4 million illnesses, 2.3 million medical visits, 58,000 hospitalizations, and 3,500 deaths (*9*).

Current influenza vaccines are providing substantial public health benefits; however, more effective influenza vaccines are needed. Therefore, many U.S. government agencies (including CDC, the National Institutes of Health, the Food and Drug Administration, and the Biomedical Advanced Research and Development Authority) are collaborating to improve influenza vaccines in support of the executive order issued by the White House on September 19, 2019.^¶¶^

Influenza antiviral medications remain an important adjunct to influenza vaccination. CDC recommends antiviral treatment for any patient with suspected or confirmed influenza who is hospitalized, has severe or progressive illness, or is at high risk for complications from influenza, including children aged <2 years and adults aged ≥65 years, regardless of vaccination status or results of point-of-care influenza diagnostic testing.*** Antiviral treatment can also be considered for any previously healthy symptomatic outpatient not at high risk for complications, with confirmed or suspected influenza, if treatment can be started within 48 hours of illness onset.

The findings in this report are subject to at least four limitations. First, sample sizes were insufficient to estimate overall VE against illnesses associated with A(H3N2) virus infections. End-of-season VE estimates could change as additional patient data become available or if a change in circulating viruses occurs later in the season. Second, vaccination status included self-report at four of five sites, which might result in misclassification of vaccination status for some patients. Third, an observational study design has more potential for confounding and bias than do randomized clinical trials. However, the test-negative design is widely used in VE studies and has been extensively validated, including against findings from randomized trials (*10*). Finally, the VE estimates in this report are limited to the prevention of outpatient medical visits rather than more severe illness outcomes, such as hospitalization or death; data from studies measuring VE against more severe outcomes this season will be available at a later date.

Annual influenza vaccination is the best strategy for preventing seasonal influenza and influenza-associated complications. This season, influenza B and A(H1N1)pdm09 viruses have cocirculated, and influenza activity remains elevated in most parts of the country. Interim VE estimates indicate that the current season’s influenza vaccine reduces the risk of medical visits associated with influenza, including visits associated with circulating influenza B viruses. Persons aged ≥6 months who have not yet received influenza vaccine during the current season should get vaccinated to protect against influenza.

**Summary**

**What is already known about this topic?**

Annual vaccination against seasonal influenza is recommended for all U.S. persons aged ≥6 months. Effectiveness of seasonal influenza vaccine varies by season.

**What is added by this report?**

According to data from the U.S. Influenza Vaccine Effectiveness Network on 4,112 children and adults with acute respiratory illness during October 23, 2019–January 25, 2020, the overall estimated effectiveness of seasonal influenza vaccine for preventing medically attended, laboratory-confirmed influenza virus infection was 45%.

**What are the implications for public health practice?**

Vaccination remains the best way to protect against influenza and its potentially serious complications. CDC continues to recommend influenza vaccination while influenza viruses are circulating in the community.

## Footnotes

* Fever (temperature ≥100°F [37.8°C]) and a cough or a sore throat without a known cause other than influenza (https://www.cdc.gov/flu/weekly/overview.htm).

† Study enrollment began at each site after local surveillance identified increasing weekly influenza activity or one or more laboratory-confirmed cases of influenza per week for 2 consecutive weeks. The U.S. Flu VE Network sites and the dates enrollment began are as follows: University of Michigan School of Public Health (partnered with the University of Michigan Health System, Ann Arbor, Michigan, and the Henry Ford Health System, Detroit, Michigan) (November 20, 2019); University of Pittsburgh Schools of the Health Sciences (partnered with the University of Pittsburgh Medical Center, Pittsburgh, Pennsylvania) (November 25, 2019); Kaiser Permanente Washington, Seattle, Washington (November 25, 2019); Marshfield Clinic Research Institute, Marshfield, Wisconsin (December 30, 2019); and Baylor Scott and White Health, Texas A&M University College of Medicine, Temple, Texas (October 23, 2019).

§ https://www.cdc.gov/flu/professionals/diagnosis/molecular-assays.htm.

¶ 100% x (1 − odds ratio [ratio of odds of being vaccinated among outpatients with CDC’s real-time RT-PCR influenza-positive test results to the odds of being vaccinated among outpatients with influenza-negative test results]).

** Sample sizes to achieve an adequate number of influenza cases to estimate a significant VE with 95% confidence intervals that do not include zero were estimated by virus subtype and the following age groups: 6 months–17 years, 18–49 years, 50–64 years, and ≥65 years. Sample size calculations were based on a type I error probability of 5% and a type II error probability of 20% (power 80%) to detect 40% VE against any influenza, 50% VE against influenza A(H1N1) or influenza B, and 30% VE against influenza A(H3N2). Assumptions about vaccination coverage varied by age group as follows: 50% for children and adolescents aged 6 months–17 years, 45% for adults aged 18–49 years, 60% for adults aged 50–64 years, and 80% for adults aged ≥65 years. These VE and coverage assumptions were made on the basis of pooled estimates from the 2012–13 through 2018–19 influenza seasons in the U.S. Flu VE Network. Age strata with insufficient influenza cases were aggregated to provide VE estimates for larger strata when possible.

†† https://www.cdc.gov/flu/about/burden/index.html.

§§ https://www.cdc.gov/flu/vaccines-work/past-seasons-estimates.html.

¶¶ https://www.whitehouse.gov/briefings-statements/statement-press-secretary-executive-order-modernizing-influenza-vaccines-u-s-promote-national-security-public-health.

*** https://www.cdc.gov/flu/professionals/antivirals/summary-clinicians.htm.
