# *Erratum:* Vol. 69, No. 7

**DOI:** 10.15585/mmwr.mm6912a7

**Published:** 2020-03-27

In the report “Interim Estimates of 2019–20 Seasonal Influenza Vaccine Effectiveness — United States, February 2020,” on page 182, in the Acknowledgments, Sonny Kim should have been listed as **Seung Jun Kim**.
